# The Effect of the COVID-19 Pandemic on Precocious Puberty

**DOI:** 10.31661/gmj.v13i.3112

**Published:** 2024-03-11

**Authors:** Mojdeh Banaei, Fatemeh Darsareh, Nasibeh Roozbeh

**Affiliations:** ^1^ Mother and Child Welfare Research Center, Hormozgan University of Medical Sciences, Bandar-Abbas, Iran

**Keywords:** COVID-19, Precocious Puberty, pandemic

## Dear Editor,

Puberty is a complex phenomenon that is influenced by endocrine changes as well as various peripheral and central signals, resulting in the emergence of secondary sexual characteristics, growth spurts, and the acquisition of reproductive competence [1]. Precocious puberty in girls is defined as the development of secondary sexual characteristics before the age of eight as a result of early activation of hypothalamic-pituitary-gonadal (HPG) axis [2]. Undoubtedly, genetic factors play a significant role in the occurrence of this process. Environmental factors such as weight, nutrition, dietary habits, physical activity, psychological factors, and exposure to electromagnetic fields (EMF) and/or endocrine-disrupting chemicals are all thought to play a role in influencing this process [3].

On the other hand, the novel severe acute respiratory syndrome coronavirus 2 (SARS-CoV-2) was identified as the cause of a cluster of atypical pneumonia cases worldwide and was regarded as one of the most important factors affecting physical and mental health [4]. According to available evidence during the Covid-19 pandemic, the incidence of mental problems such as depression, anxiety, and stress in children and adolescents has been reported to varying degrees as in adults [5]. School closures, sudden disruptions in social and familial relationships, changes in daily routines, and parental anxiety about financial and other problems may all have an impact on children’s emotional stability and sense of security during this pandemic [6]. All of the aforementioned factors will play a role in the occurrence of precocious puberty.

According to data from an Italian girls study, the rate of precocious puberty in girls was significantly higher at the start of the Covid-19 pandemic, compared to the same 6-month period in 2019 [7]. People’s lifestyles changed dramatically during the Covid-19 epidemic, particularly in the early stages of quarantine. Families were forced to stay at home following the Covid-19 pandemic and the closure of schools and sports activities. They had greater access to consume high-calorie foods. On the other hand, as education has become more virtualized in schools, children’s use of electronic devices such as tablets, mobile phones, or personal computers has increased. This problem led to sedentary behaviors in children and teenagers [7].

Excessive use of electronic devices for educational or recreational purposes can reduce physical activity and contribute to obesity. In addition, it causes changes in melanin levels, which are necessary for the onset of pubertal events [8]. A recent Italian study found that girls who began precocious puberty during the pandemic used electronic devices for longer periods and had lower levels of melatonin [9]. Umano et al, on the other hand, found no difference in the duration of use of electronic devices and obesity between girls with central precocious puberty (CPP) and the control group [10].

The findings of an Indian study on precocious puberty in boys and girls during the Covid-19 pandemic revealed that boys and girls had a threefold increase in referrals to endocrinology centers, with girls being more noticeable. One of the limitations of the studies on the relationship between precocious puberty and Covid-19 was that the majority of them were conducted on the female population. Precocious puberty in boys is less common and is frequently caused by organ factors. However, the studies on the role of environmental factors in boy puberty are insufficient[11]. Although this issue has been confirmed in a few studies, it is preferable to conduct more studies using prospective methods on boys and girls in pre-puberty and puberty ages to get a better explanation of this issue, so that more comprehensive results can be obtained, especially in cases of other viral infections and their effect on the puberty process.

## Conflict of Interest

None.
